# Adenovirus-36 Is Associated with Obesity in Children and Adults in Sweden as Determined by Rapid ELISA

**DOI:** 10.1371/journal.pone.0041652

**Published:** 2012-07-27

**Authors:** Malin Almgren, Richard Atkinson, Jia He, Agneta Hilding, Emilia Hagman, Alicja Wolk, Anders Thorell, Claude Marcus, Erik Näslund, Claes-Göran Östenson, Martin Schalling, Catharina Lavebratt

**Affiliations:** 1 Department of Molecular Medicine and Surgery, Karolinska Institutet, Stockholm, Sweden; 2 Center for Molecular Medicine, Karolinska University Hospital, Stockholm, Sweden; 3 Department of Clinical Neuroscience, Karolinska Institutet, Stockholm, Sweden; 4 Obetech Obesity Research Center and Virginia Commonwealth University, Richmond, Virginia, United States of America; 5 Division of Pediatrics, Department of Clinical Science, Intervention and Technology, Karolinska Institutet, Stockholm, Sweden; 6 Division of Nutritional Epidemiology, Institute of Environmental Medicine, Karolinska Institutet, Stockholm, Sweden; 7 Department of Clinical Sciences, Danderyd Hospital, Karolinska Institutet and Department of Surgery, Ersta Hospital, Stockholm, Sweden; 8 Division of Surgery, Department of Clinical Sciences, Danderyd Hospital, Karolinska Institutet, Stockholm, Sweden; University of Ottawa, Canada

## Abstract

**Background:**

Experimental and natural human adenovirus-36 (Adv36) infection of multiple animal species results in obesity through increasing adipogenesis and lipid accumulation in adipocytes. Presence of Adv36 antibodies detected by serum neutralization assay has previously been associated with obesity in children and adults living in the USA, South Korea and Italy, whereas no association with adult obesity was detected in Belgium/the Netherlands nor among USA military personnel. Adv36 infection has also been shown to reduce blood lipid levels, increase glucose uptake by adipose tissue and skeletal muscle biopsies, and to associate with improved glycemic control in non-diabetic individuals.

**Principal Findings:**

Using a novel ELISA, 1946 clinically well-characterized individuals including 424 children and 1522 non-diabetic adults, and 89 anonymous blood donors, residing in central Sweden representing the population in Stockholm area, were studied for the presence of antibodies against Adv36 in serum. The prevalence of Adv36 positivity in lean individuals increased from ∼7% in 1992–1998 to 15–20% in 2002–2009, which paralleled the increase in obesity prevalence. We found that Adv36-positive serology was associated with pediatric obesity and with severe obesity in females compared to lean and overweight/mildly obese individuals, with a 1.5 to 2-fold Adv36 positivity increase in cases. Moreover, Adv36 positivity was less common among females and males on antilipid pharmacological treatment or with high blood triglyceride level. Insulin sensitivity, measured as lower HOMA-IR, showed a higher point estimate in Adv36-positive obese females and males, although it was not statistically significant (p = 0.08).

**Conclusion:**

Using a novel ELISA we show that Adv36 infection is associated with pediatric obesity, severe obesity in adult females and lower risk of high blood lipid levels in non-diabetic Swedish individuals.

## Introduction

Human adenovirus-36 (Adv36) was first isolated in 1980 [1] and belongs to the group of 54 known human adenovirus serotypes generally associated with infections in the respiratory or gastrointestinal tract or the conjunctiva. The 54 types are grouped into seven species based on their immunochemical responses, nucleic acid characteristics, hexon and fiber protein characteristics, biological properties, and phylogenetic analysis, with Adv36 belonging to the adenovirus-D subgroup (Adv-D) [2], [3]. It has been demonstrated that experimental and natural Adv36 infection of multiple animal species resulted in obesity through increasing proliferation and differentiation of preadipocytes and lipid accumulation in mature adipocytes [4]–[12]. Most subsequent papers on Adv36 and obesity in man have shown that humans with evidence of prior, natural Adv36 infection are heavier than uninfected individuals [13]–[19]. The data on association between Adv36 and obesity in adults differ between studies being somewhat inconsistent, but the findings in children consistently associate Adv36 infection with obesity.

In contrast to most cases of obesity, Adv36-associated obesity has been associated with lower levels of blood lipids in animal [5] and adult man [13], [14]. Also, Adv36 is reported to improve glycemic control in rat [6] and in chow-fed and in high-fat diet-fed mice by enhancing glucose uptake in adipose tissue, skeletal muscle and reduce hepatic glucose release [20]–[22]. Further, Adv36 was associated with enhanced insulin sensitivity in 1507 lean, overweight and obese otherwise healthy adults and children of different ethnicities living in the USA [22].

All of the studies published previously used the serum neutralization assay (SNA) to detect antibodies against Adv36. The SNA is quite time consuming and expensive, taking approximately 2 weeks to perform. Hence, it is not well suited for rapid screening for Adv36 infection.

In this paper we have described a new method for determining evidence of Adv36 infection. Using the ELISA we tested the hypotheses that Adv36 was present within the Swedish population living in the area of the capital Stockholm, that Adv36 was associated with obesity in children and adults in Stockholm area, that Adv36 was associated with lower blood lipid levels, and that Adv36 was associated with insulin sensitivity in non-diabetic individuals.

## Materials and Methods

### Ethics Statement

A total of 424 children and 1522 adults from Sweden, and 456 anonymous human serum samples from Sweden, the USA, Finland and South Korea, were studied in this project. The study groups and the studies they were used in are listed in Table 1. This project is in compliance with the Code of Ethics of the World Medical Association (Declaration of Helsinki). The ethical committee of Karolinska Institutet approved the study and informed consent was obtained from all participants, but for the Swedish commercial anonymous blood donor samples. The informed consent was written, but for in the Stockholm Diabetes Prevention Program (SDPP). For participants in SDPP, verbal informed consent was obtained according to a procedure approved by the ethics committee. This was documented in the SDPP research protocol, a procedure fulfilling the Swedish legal requirements. Also the guardians of participating children (age below 16 years) gave written consent.

**Table 1:** Study groups and the studies they were used for.

Study group	City	Age (years)	Year collected	Lean (n)	Overweight/obese (n)	Study
Pediatric obesity patients	Stockholm	10–18	2003–2007		113 f, 108 m	3a
High school students	Stockholm	16–17	2004–2006	106 f, 97 m		2, 3a
Obesity surgery clinic	Stockholm	19–67	2008–2010		241 f	3b
SDPP	Stockholm	35–55	1992–1998	125f, 55m		2
SDPP	Stockholm	45–65	2002–2006	255 f, 219 m	252 f, 218 m	2,3b
SMC	Uppsala	55–80	2004	81 f	76 f	2,3b
Anonymous blood donors	Stockholm	19–69	2009		55 f, 34 m	2
Anonymous samples	USA, Finland, South Korea				367	1

f: females, m: males.

Study 1: Comparison between ELISA and serum neutralization assay.

Study 2: Adv36 prevalence in Sweden.

Study 3: Association analyses between Adv36 and metabolic parameters in a) children and b) adults.

The vaccination of rabbits was performed in strict accordance with the recommendations in the Guide for the Care and Use of Laboratory Animals of the National Institutes of Health. The protocol was approved by the Committee on the Ethics of Animal Experiments of the University of Wisconsin.

**Figure 1:**
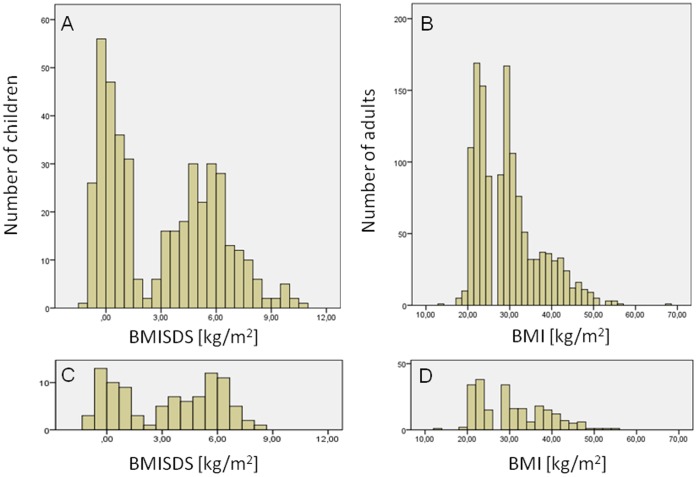
Distribution of BMI in analyzed children and adults. A) Distribution of BMI SDS (BMI Z-Score) adjusted for age and gender according to Rolland-Cachera et al, 1982 [24] among lean (BMI SDS: −1.4−1.8) and overweight/obese (BMI SDS: 2.2–10.5) children; B) Distribution of BMI in the lean (BMI<25 kg/m^2^), overweight and mildly obese (28<BMI<35 kg/m^2^), and severely obese (BMI≥35 kg/m^2^) adults studied; C) BMI-SDS distribution of those children positive for Adv36 in ELISA; D) BMI-distribution of those adults positive for Adv36 in ELISA.

### Obese Children

Children aged 10–18 years and referred to the national pediatric obesity clinic center at Karolinska University Hospital in Huddinge, Stockholm County, were studied. The children studied can be regarded as representing pediatric obesity patients in the Stockholm County population because children in Stockholm County seeking health care for obesity would be referred to this clinic. Serum and clinical data from the 221 obese patients, including 113 girls and 108 boys, were collected between 2003 and 2007 (Table 1). Date of serum collection and date of clinical data collection for each patient occurred within 6 months. The vast majority (71%) of the children were sampled before any treatment of obesity, whereas for the rest (29%) information on treatment prior to sampling was unavailable. Most of the children were obese (101 females and 98 males) and a few were overweight (12 females and 10 males) according to international cutoff limits [23]. The distribution of BMI SDS for these children had a median (25^th^, 75^th^ percentiles) of 5.5 (4.4, 6.4) and ranged between 2.2 and 10.5 [24] (Fig. 1). (BMI SDS, or BMI Z-Score, is the number of standard deviations the child’s BMI is from French population BMI mean adjusted for the child’s age and gender according to Rolland-Cachera et al, 1982 [24]). Clinical data available for all patients included levels for fasting plasma triglycerides, total cholesterol, LDL, HDL, glucose and insulin. These levels were measured by the Karolinska University Hospital’s accredited chemistry laboratory. Insulin sensitivity (Si) and acute insulin response (AIR), obtained by means of frequent sampling insulin modified glucose tolerance test [25], were available for the 71% of individuals known not to have had prior treatment of obesity (Table 2).

**Table 2:** Clinical characteristics of the study groups.

	Obese children	SDPP and SMC females and males			Obesity surgery clinic females
		BMI<25 kg/m^2^	28≤BMI<35 kg/m^2^	BMI≥35 kg/m^2^	BMI≥35 kg/m^2^
n_females_, n_males_	113, 108	336, 219	301, 207	27, 11	241, 0
Antilipid drug [n_yes_/n_no_]		15/459	27/405	1/37	12/229
Total cholesterol [mM]^a^	4.0 (3.4, 4.5)^p^	6.3 (5.6, 7.0)	6.4 (5.9, 6.9)	6.4 (5.6, 7.9)	4.6 (4.0, 5.4)^p^
LDL [mM]	2.6 (2.0, 3.1)^p^				
HDL [mM]	1.0 (0.80, 1.2)^p^				
Triglycerides [mM]^a^	1.1 (0.70, 1.6)^p^	1.0 (0.87, 1.6)	1.6 (1.2, 2.3)	2.1 (1.4, 3.3)	1.2 (0.89, 1.5)^p^
Glucose [mM]	5.2 (4.9, 5.5)^p^	4.7 (4.4, 5.0)	5.0 (4.7, 5.3)	5.0 (4.7, 5.6)	5.3 (5.0, 5.6)^p^
Insulin [µU/ml]	14.4 (9.9, 21.5)^p^	12.0 (10.0, 15.0)	16.0 (13.0, 19.0)	22.0 (16.5, 27.0)	9.5 (6.2, 14.0)
HOMA-IR^b^		2.5 (2.0, 3.2)	3.7 (2.8, 4.6)	4.8 (3.9, 6.8)	2.2 (1.4, 3.4)
Insulin sensitivity (Si)	2.3 (1.5, 3.9)				
Acute insulin response (AIR)	893 (589, 1470)				

No individual had diabetes. The lean children group is not included.

Values are median (25^th^,75^th^ percentile). SMC samples (81 lean females (BMI<25 kg/m^2^) and 76 overweighed/mildly obese females (28≤BMI<35 kg/m^2^)) had no data for these characteristics but are included in the total n. For SDPP, blood lipid data was available from 172 lean, 103 overweighed/mildly obese and 9 severely obese individuals. Other data was available from all SDPP individuals. The lean children group is not included here since these characteristics were unknown for them.

a Those on pharmacological treatment for high blood lipid levels (statins or fibrates) were excluded.

b HOMA-IR was not analyzed in children because limited correlation with Si in children [41].

p Fasting plasma levels. Otherwise, data are fasting serum levels.

### Lean Children

Two hundred-three lean controls aged 16–17 years were collected from high schools in Stockholm between 2004 and 2006 (Table 1). The distribution of BMI SDS for these lean children had a median (25^th^, 75^th^ percentiles) of 0.2 (−0.2, 0.8) and ranged from −1.4 to 1.8 [24] (Fig. 1). Serum and data on age, gender, body weight, height, and school name were available. Schools with a proportion higher than average of children with non-Swedish origin were excluded. Serum collection and clinical data collection for each child occurred on the same day.

### Adult Severely Obese Female Patients from Obesity Surgery Clinics in Stockholm

Females referred to the obesity surgery clinics at Danderyd Hospital and Ersta Hospital in Stockholm for obesity treatment between 2008 and 2010 were studied. Those with BMI≥35 kg/m^2^, no known molecular cause of obesity but with data on fasting plasma triglycerides, total cholesterol, glucose and serum insulin were included. Females with T2DM were excluded. To exclude those with prediabetes, HOMA-IR<6.6 was used as an arbitrary criterion for inclusion. In total, 241 females aged between 19 and 67 years (age; mean ± SEM: 40.4±0.63 years) were included in the study (Table 1). Serum and data on fasting plasma triglycerides, total cholesterol, glucose, fasting serum insulin, and HOMA-IR ( =  glucose*insulin/22.5, reflects insulin resistance) were used (Table 2). Analyses of glucose, insulin, cholesterol and triglycerides were performed by the Karolinska University Hospital’s accredited chemistry laboratory. In short, glucose (reference (normal) interval 4.0–6.0 mmol/L) was analyzed on a Beckman Coulter LX (Beckman Coulter AB, Bromma, Sweden), insulin (reference interval 2.6–25.9 µU/ml) on a Modular E170 (Roche Diagnostics AB, Bromma, Sweden), cholesterol (reference interval plasma and serum for age 31–50 years: 3.3–6.9 mmol/l; >50 years: 3.9–7.8) and triglycerides (reference interval plasma and serum for age >18 years: 0.45–2.6 mmol/l) on a Beckman Coulter DXC800 (Beckman Coulter AB).

### Adult Obese, Overweight and Lean Female and Male Individuals from the General Stockholm Population

#### The Stockholm Diabetes Prevention Program (SDPP)

Individuals selected from the longitudinal population-based SDPP cohort of females and males living in Stockholm County, aged 35–56 years at baseline, were studied. The baseline sampling was performed in 1992–1998 and follow-up was performed in 2002–2006 (Table 1). A small set of serum samples collected at baseline was analyzed for prevalence of Adv36 antibodies 1992–1998: 125 females (age; mean ± SEM: 46.2±5.4 years) and 55 males (age; mean ± SEM: 47.0±4.9 years) that were lean (BMI 20–24.9 kg/m^2^) at baseline with fasting serum glucose <6.1 mM and normal oral glucose tolerance defined as glucose level <7.8 mM 2 h after 75 g oral glucose tolerance test (OGTT) [26]. For Adv36 prevalence analysis 2002–2006 and analysis of Adv36 association to obesity, serum and data used in this study were from the follow-up collected in 2002–2006. Individuals with BMI≥28.0 kg/m^2^ were selected to the overweight/mild obesity group consisting of 218 males (age; mean ± SEM: 56.2±0.33, range 46–66 years) and 252 females (age; mean ± SEM: 55.5±0.32, range 44–63 years). A lean group (BMI 20.0–24.9 kg/m^2^) was matched by age and sex to the overweight/mild obesity group and included 219 males (age; mean ± SEM: 56.8±0.34, range 46–65 years) and 255 females (age; mean ± SEM: 55.6±0.32, range 44–63 years). All selected individuals had normal oral glucose tolerance and normal fasting serum glucose levels at both baseline and follow-up. Serum and clinical data on BMI, fasting serum triglycerides, total cholesterol, glucose, insulin and HOMA-IR were used (Table 2). Serum and clinical data were collected at the same date for each individual. Concentration of serum glucose was analyzed in duplicate by a glucose oxidase method using a Yellow Springs Glucose Analyzer (Yellow Springs, OH, USA). Serum insulin was assayed by radioimmunoassay, using in-house antibodies, human insulin as a standard, and charcoal addition to separate antibody-bound and free serum insulin [27]. Cholesterol and triglyceride levels were measured by the Karolinska University Hospital’s accredited chemistry laboratory as described for the obesity surgery clinical patients.

#### The Swedish Mammography Cohort (SMC)

Adult females selected from the population-based The Swedish Mammography Cohort (SMC) females in Uppsala County were studied for Adv36 antibodies. From 1987 to 1990, 74% of all females living in Uppsala County and born between 19141948 returned a completed questionnaire on diet, weight, height and education. During 2003–2009 a subgroup of those (5022 females) participated in a physical examination after an overnight fast where BMI, plasma and serum were obtained. For this study, from the 5022 females 76 overweight/obese (BMI≥28.0 kg/m^2^) and 81 lean (BMI  = 20.0–24.9 kg/m^2^) non-diabetic females examined in 2004 were randomly selected (Table 1). Serum and clinical data were collected at the same date for each individual. No metabolic blood chemistry data was used.

Combining females from SDPP and from SMC there were in total 328 overweight/obese females (age; mean ± SEM: 57.8±0.37 years) and 336 lean females (age; mean ± SEM: 57.8±0.36 years) with age range 44–80 years.

#### Anonymous blood donors

Serum was collected in 2009 from 89 healthy anonymous blood donors at Karolinska University Hospital in Huddinge. They were 34 males and 55 females aged 19–69 years, median age 40 years (Table 1).

### Serum Neutralization Assay (SNA) for Viral Antibodies

Positive control serum samples were produced by inoculating three rabbits with killed Adv36 emulsified in Freund’s adjuvant via intramuscular injection and collection of blood 29 days post-injection (Obetech, Richmond,VA). Human serum samples (n = 367) from the United States, Korea and Finland (Table 1) were used to compare the Adv36-ELISA to the Adv36-serum neutralization assay (SNA). The SNAs were performed, as described previously [4], [5] (see information S1). In brief, human sera were serially diluted (two-fold) from 1∶2 to 1∶128 in 96-well plates, whereas the rabbit sera were diluted from 1∶40 to 1∶10240. Each serum sample and the positive rabbit control serum were run in duplicate with additional appropriate controls [4], [5]. Serum samples without CPE in dilutions of 1∶8 or higher were considered positive. Disparate readings with one reading above and one below the cutoff value were considered equivocal.

### ELISA for Antibodies Against Adv36

The investigation is presented according to the Standards for the Reporting of Diagnostic Accuracy (STARD) (http://stard-statement.org/). The analyses were performed by researcher blinded to any phenotypic and SNA data. 96-well ELISA microplates were coated overnight at 4°C with recombinant Adv36 fiber protein fragment fused with maltose binding protein (10 µg/ml, Obetech, Richmond, VA). After washing step, blocking with 20 mg/ml of bovine serum albumin for 1 h, and another washing step, a mixture of horse radish protein (HRP conjugated coating protein, Obetech, Richmond, VA) and the competitor protein recombinant maltose binding protein (62.5 µg/ml, MBP, Obetech, Richmond, VA) as well as serum was added to each well. Each 96-well plate had one positive serum control (50 µl positive rabbit control serum (diluted 1∶80) (Obetech, Richmond, VA)), one background control (50 µl of blocking buffer), and at least one control serum known to be at the cutoff value for positive ELISA score being human serum (50 µl) and rabbit serum (50 µl of dilution 1∶1280). After 1 h of incubation and subsequent washing, TMB substrate solution (3,3′,5,5′ – tetramethylbenzidine, Thermo Scientific 34028, Rockford, IL) was added to each well, and reaction stopped after ∼25 min with 1M HCl. The plate was read at a wavelength of 450 nm. All samples were run in duplicate. The positive serum control must have an optical density (OD_450_) reading greater than 1.5, and the background control must have a reading below 0.06 or the assay was invalid. The negative human control serum should be within OD_450_±0.05 from its reference value. ELISA cutoff for positive score was based on analysis of human serum samples with known Adv36-SNA status. Thereafter, the OD_450_ reading from the 1∶1280 dilution of the positive rabbit serum was used as cutoff marker for positive ELISA score since it gave a signal corresponding to the defined ELISA cutoff. An OD_450_ equal or greater than this value in both duplicates was scored as a positive assay for Adv36 antibodies. A reading of less than this value in both duplicates was scored negative. Disparate readings with one reading above and one below the cutoff value were considered equivocal and the sample was reanalyzed in duplicate. A sample with repeat analyses was considered to be positive if >50% of the replicates were positive with an OD_450_ higher than the cutoff value. Hence, data from a reanalyzed serum sample were used only if the reanalysis provided consistency between the duplicates. Human serum samples (n = 367) from United States, Korea and Finland were used to compare the Adv36-ELISA scores to the Ad36-SNA scores. These samples were not reanalyzed. For protocol details see information S1.

### Statistical Analysis

In the children Adv36 positivity in the ELISA was analyzed for statistically significant association to obesity using the Pearson’s chi-square test, along with estimation of odds ratio (OR) and 95% confidence interval (CI), and using logistic regression including Adv36 status, sex and age as covariates. For the children the following quantitative clinical outcome variables were analyzed with regard to Adv36 status: BMI SDS [24], levels of fasting plasma triglycerides, total cholesterol, LDL, HDL, glucose, insulin, Si and AIR. To normalize the distribution of the quantitative variables they were transformed by the natural logarithm (ln). The association analyses were performed using ANCOVA with Ad36 status, age and sex as independent factors. Also BMI SDS was included as independent factor for outcome variables other than BMI SDS. The distribution of the residuals was normal.

For the adults, association between Adv36 positivity and obesity was tested in each sex separately using the Pearson’s chi-square test, and logistic regression with Adv36 and age as covariates. Association between Adv36 status and marker for high levels of circulating blood lipids, i.e. being on pharmacological treatment for high blood lipid level and/or having high triglyceride level, was tested using Pearson’s chi-square test, and logistic regression with Adv36, cohort ID, BMI group, sex and age as covariates. Individuals known to not be on treatment but missing data on blood triglyceride levels were excluded from this analysis. A circulating triglyceride level above the normal interval (0.45–2.6 mmol/l), i.e. >2.69 mmol/l was regarded as high. The quantitative clinical variables for adults analyzed for association to Adv36 status were BMI, waist circumference, levels of fasting levels of triglycerides, total cholesterol, glucose, insulin and HOMA-IR (higher level reflecting higher insulin resistance). The quantitative variables were transformed by the natural logarithm (ln) and association between Adv36 positivity and clinical data was analyzed using unpaired *t*test and ANCOVA with age and BMI as covariates for normally distributed variables, and using Mann-Whitney U tests for non-normally distributed variables. If samples from SDPP and clinical patients were combined in the quantitative outcome variable analysis, ANCOVA was applied using age, cohort-ID and BMI as additional covariates. The distribution of the residuals was normal. If female and male samples were included in the same analysis, sex was used as additional covariate in the ANCOVA. For quantitative analysis of triglyceride and cholesterol levels, individuals on pharmacological treatment for high blood lipid levels were excluded. All analyses were performed using SPSS version 19. Reported p-values are two-tailed. A two-tailed p-value below 0.05 was regarded as significant.

## Results

The study groups used within the different studies (study 1, 2, 3a and 3b) are listed in Table 1.

### An ELISA for the Detection of Antibodies Against Human Adenovirus-36 (Study 1)

An ELISA detecting antibodies against a recombinant Adv36 coat protein (the fiber protein) in serum was developed. A total of 367 human sera from five sets of samples from different geographical areas were used to compare this ELISA with the standard method, the serum neutralization assay (SNA). For some of the sets, individual serum samples were selected based on prior SNA results, so the frequency of antibody positive tests did not reflect population Adv36 infection prevalence. The assays were run with the researcher blind to the results of the other assay. Table 3 shows the comparison between the ELISA and the SNA. Of the samples positive in the SNA, 74.3% were positive also in the ELISA (Standard error of proportion (SEP)  = 7.4; 26 ELISA-positive of 35 SNA-positive samples), and of the samples negative in the SNA 63.1% were negative also in the ELISA (SEP  = 2.8; 185 ELISA-negative of 293 SNA-negative samples). The proportion of samples with equivocal scoring was lower in ELISA (3.3%) compared to in SNA (7.6%) (OR = 0.4, p = 0.015). The between-duplicate correlation of Adv36-ELISA OD_450_ values was very good (r = 0.996, n = 367, Pearson’s correlation coefficient).

**Table 3:** Comparison between the ELISA and the serum neutralization assay (SNA) for Adv36 antibody detection.

	ELISA-negative	ELISA-positive	ELISA-equivocal	Totals
SNA-negative	185	108	9	302
SNA-positive	9	26	2	37
SNA-equivocal	16	11	1	28
Totals	210	145	12	367

**Figure 2:**
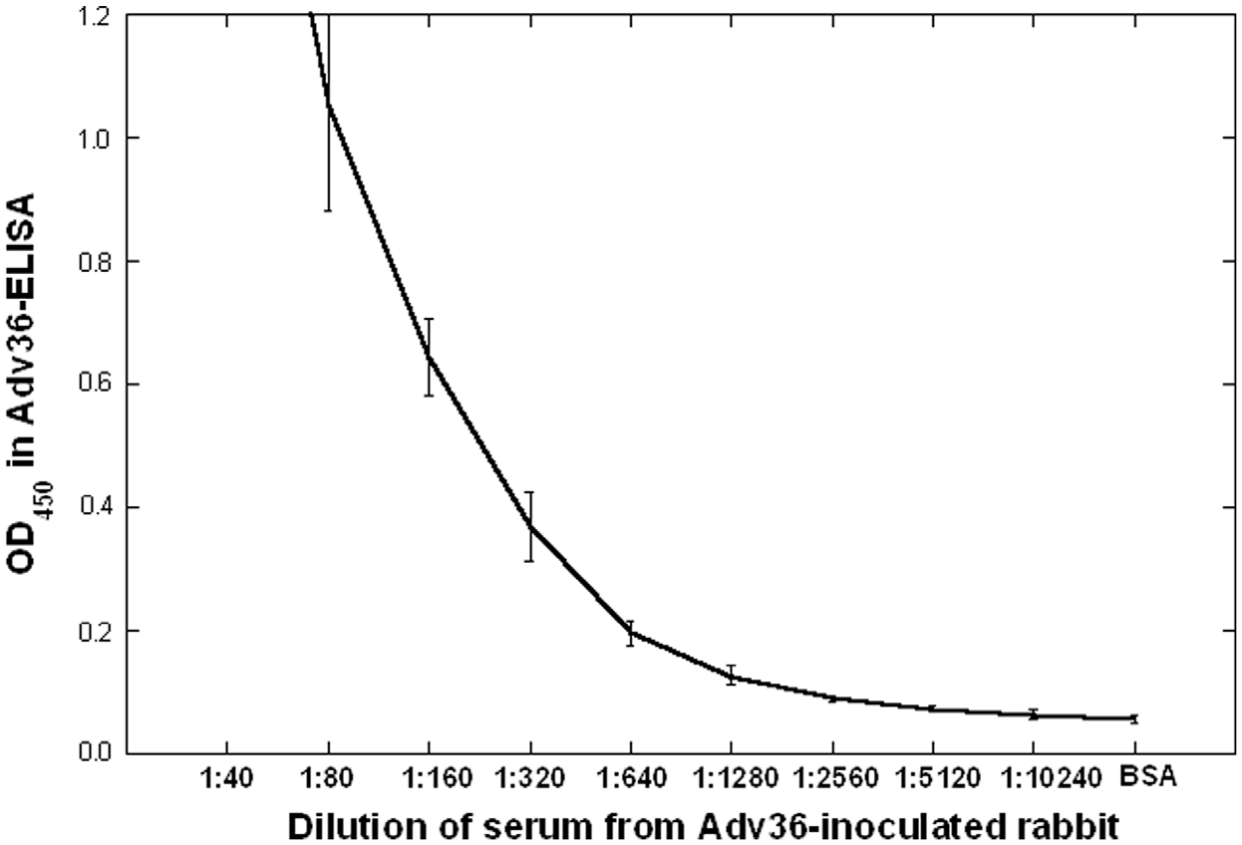
ELISA titration curve. ELISA titration curve of serum from rabbit inoculated with Adv36. Cutoff for positivity in the ELISA is at rabbit serum dilution 1∶1280 (OD_450_ = 0.12), that is in the low linear region. The average optical density at 450 nm of three experiments, each in duplicate, from one rabbit is shown. Error bars indicate SEM. Similar data were obtained from two additional rabbits.

Data from rabbit serum samples suggested that the higher rate of seropositivity in the ELISA in comparison to the SNA might in part be due to the ELISA detecting lower titers of Adv36-antibodies than the SNA did. Serum samples collected from rabbits inoculation with killed Adv36 were analyzed in parallel using the ELISA and the SNA. The titration curve (steps of 1∶2) showed that the ELISA was positive down to dilution 1∶1280 (OD_450_≥0.12) (Fig. 2) whereas the SNA did not detect Adv36 antibodies in dilutions greater than 1∶160 (data not shown).

There is a high degree of homology of proteins among adenoviruses, for Adv36 especially to other adenoviruses belonging to group Adv-D [3]. Although the ELISA-based antigen was selected to be as specific as possible, cross reactivity with other non-Adv36 adenoviruses may exist. The extent to which the Adv36-ELISA cross reacted with two other Adv-D adenoviruses, human adenovirus-37 (Adv37) and human adenovirus-9 (Adv9), were roughly estimated. Thirty-one samples that were negative for Adv36 by Adv36 SNA were evaluated for antibodies to Adv37 and for antibodies to Adv9 by their respective serum neutralization assays. Of the 31 samples, 11 were positive for any or both of Adv37 and Adv9 (one for Adv37 alone, five for Adv9 alone, and five for both Adv37 and Adv9). Of these 11 samples, 5 were positive for Adv36 by ELISA (Table 4). Four of the 6 Adv37-positive samples were positive in the ELISA. While this may indicate cross reactivity of the ELISA with Adv37 and potentially explain some non-specificity of the ELISA versus Adv36 SNA, the rate of ELISA-positivity in relation to positivity of Adv37 and Adv9 SNAs (5 of 11 = 45%) was not significantly different to that of ELISA-positivity among samples negative for Adv36 SNA (108 of 293 = 37%) (p = 0.75).

**Table 4:** Proportion Adv36-ELISA-positive samples among those scoring negative or positive for Adv37 and/or Adv9.

	Adv9-negative	Adv9-positive	Adv9-equivocal	Totals
Adv37-negative	9/17	1/5	2/3	12/25
Adv37-positive	0/0	4/5	0/1	4/6
Adv37-equivocal	0/0	0/0	0/0	0/0
Totals	9/17	5/10	2/4	16/31

All samples scored negative for Adv36 in the serum neutralization assay.

The average exclusion rate, i.e. the average proportion of samples being “equivocal”, in the 9 sample BMI-groups (children and adults) reported below was 3.9%. The exclusion rate was not significantly different between the BMI-groups (p>0.3).

### Antibodies Against Human Adenovirus-36 are Present in Serum from Adults and Children in Sweden (Study 2)

**Figure 3:**
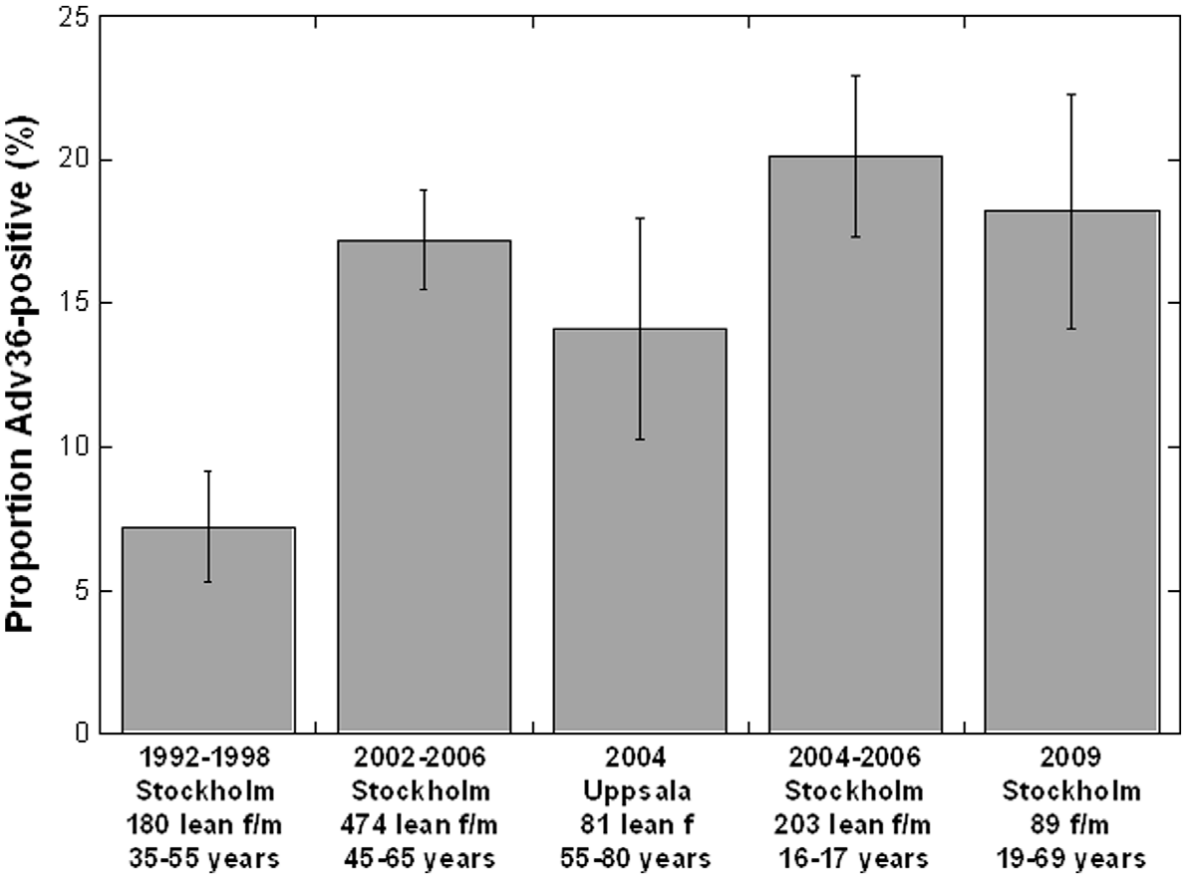
Prevalence of Adv36 in lean Swedes. Prevalence of positive Human adenovirus-36 serology in serum samples from adults and children living in Stockholm and Uppsala, Sweden, between 1992/1998 and 2009. The Adv36 serology was determined using the Adv36-ELISA. Error bars indicate standard error of proportion.

The Adv36-ELISA was used to analyze serum samples from the population-based cohorts SDPP and SMC from the adult Stockholm and Uppsala population, serum samples from anonymous blood donors from Stockholm and serum samples from lean children (16–17 years old) from Stockholm. Serum samples collected between 1992 and 1998 from 180 lean (BMI<25 kg/m^2^) adults living in Stockholm County and aged 35–55 years, selected from SDPP, had a prevalence of Adv36-positive serology of 7.2% (Fig. 3). Serum samples collected in 2002–2006 from lean adults in Stockholm, 219 males and 255 females aged 46–66 years selected from SDPP, had a prevalence of 15.6% among females and 19.5% among males, with combined prevalence of 17.6% in males and females. Serum collected 2004 from 81 lean (BMI<25 kg/m^2^) postmenopausal females in Uppsala, selected from SMC, had an Adv36-positive serology prevalence of 14.1%. Also serum samples from 203 lean children aged 16–17 years from high-schools in Stockholm collected 2004–2006 were analyzed for prevalence of Adv36-positive serology. Similar to the prevalence in adults 2002–2006, the children had a prevalence of 20.1%. Hence, the prevalence of Adv36 in Stockholm increased from ∼7% in 1992–1998 to 15–20% in 2002–2006. To estimate the prevalence of Adv36 positivity prevalence in 2009, serum samples were collected in 2009 from 89 healthy anonymous blood donors in Stockholm (34 men and 55 females aged 19–69 years) and analyzed for Adv36 serology. The prevalence of positive Adv36 serology was 18.2%. Hence, marker for ongoing or past Adv36 infection was present in the adults and children population in the Stockholm-Uppsala area of Sweden. The prevalence of Adv36 in Stockholm increased from ∼7% in 1992–1998 to 15–20% in 2002–2006, and was stable until 2009. The prevalence of positive Adv36 serology was similar in adults and children.

### Adv36 is More Common in Obese than Lean Swedish Children (Study 3a)

**Figure 4:**
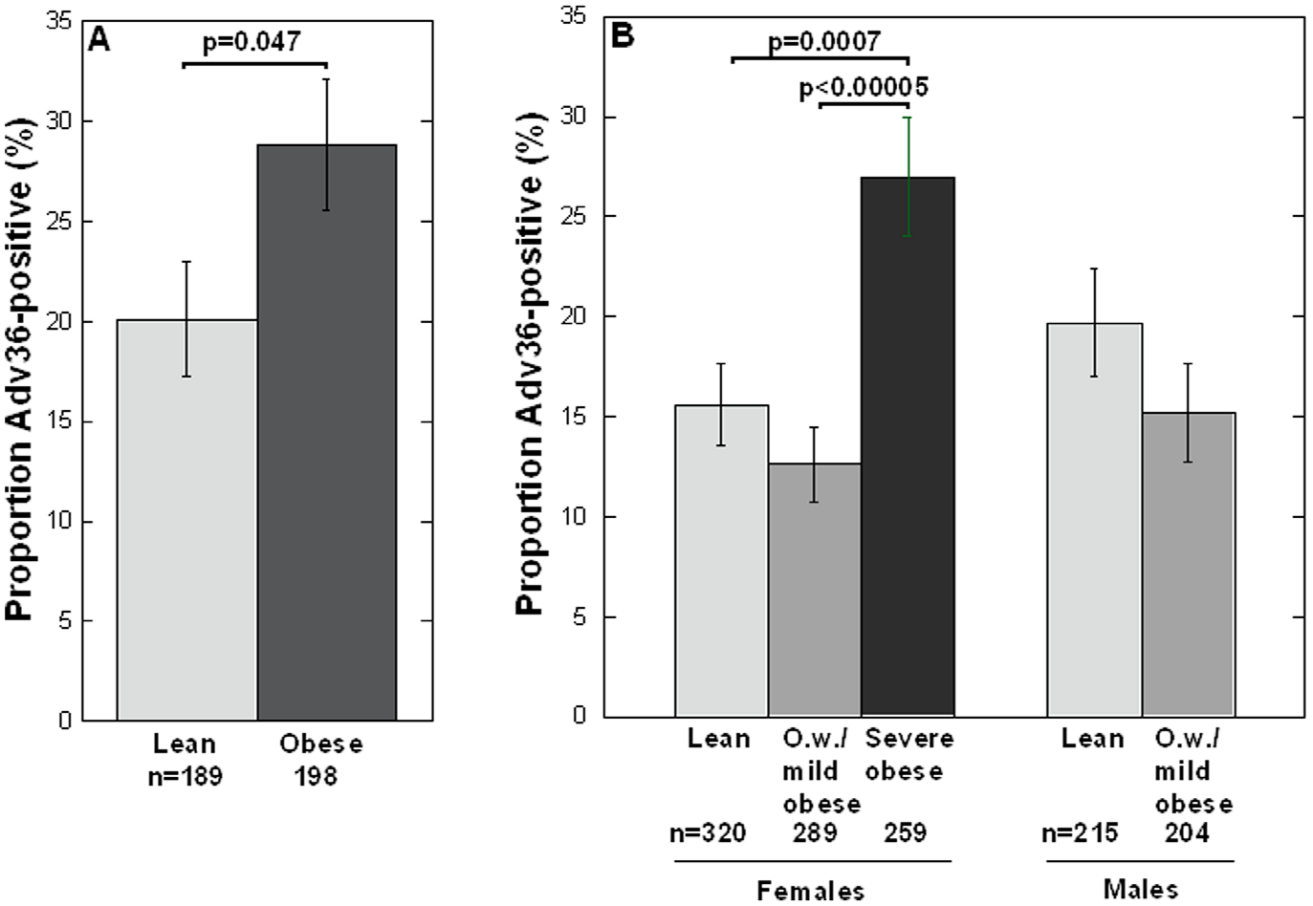
Association between Adv36 and obesity. A) Higher proportion of children with positive Adv36 serology among pediatric obesity patients (BMI SDS: 2.2–10.5) than lean (BMI SDS: −1.4−1.8) children from high schools in Stockholm. B) Higher prevalence of positive Adv36 serology among severely obese (BMI≥35 kg/m^2^) females compared to lean (BMI<25 kg/m^2^) and overweight/mildly obese (O.w./mild obese) (28<BMI<35 kg/m^2^) females in Stockholm and Uppsala. Error bars indicate standard error of proportion. Due to a low number of severely obese males (n = 11), Adv36 association to severe obesity in males could not be analyzed. However, no difference in Adv36 positivity was found between females and males in lean and overweight/mildly obese groups, and no difference in prevalence of Adv36 positivity was detected among males between lean and overweight/mildly obese.

Children referred to pediatric obesity clinic for obesity as well as lean controls from high schools (Fig. 1), all cases and controls being from Stockholm County, were analyzed for Adv36 antibodies in serum using ELISA. The obese/overweight children had a higher prevalence of positive Adv36 serologic status (28.8%) compared to the lean controls (20.1%) (Fig. 4A). Hence, Adv36 positivity was statistically more common in obese/overweight than in lean children (OR = 1.6, 95% CI: 1.0–2.6; χ^2^(1) = 3.9, p = 0.047). This was found also by a logistic regression analysis where Ad36 status, sex and age were used as covariates (Adv36 status: OR = 1.7, 95% CI: 1.0–2.9, p = 0.050; age: b = −0.78, SEM: 1.8, p<0.001; sex: p>0.1) (Table 5). Also, the quantitative BMI SDS variable was borderline linearly dependent on Adv36 status in the obese/overweight and lean children (ln(BMI SDS): unstandardized b = 0.21, SEM  = 0.11, F = 3.4, p = 0.064; age: b = −0.20, SEM  = 0.021, F = 87.3, p<0.001, ANCOVA). Blood lipid levels (fasting plasma triglycerides, total cholesterol, HDL, LDL) were not different between Adv36-positive compared to Adv36-negative obese/overweight children (p>0.3), and there was no effect of Adv36 positivity on glucose, insulin, insulin sensitivity (Si) or AIR among these samples (p>0.3).

**Table 5:** Association for Adv36 positivity to obesity.

Study group	Adv36pos	Adv36neg	χ^2^, p-value	χ^2^, p-value
	% (n)	% (n)	*vs* BMI<25 kg/m^2^	*vs* 28≤BMI<35 kg/m^2^
*Children*				
Overweight/obese (BMI SDS: 2.2–10.5)^a^	28.8 (57)	71.2 (141)	3.9, 0.047	
Lean (BMI SDS: −1.4−1.8)^b^	20.1 (38)	79.9 (151)		
*Females*				
Severely obese (BMI≥35 kg/m^2^)^c^	28.4 (66)	71.6 (166)	13.5, 0.0002	19.9, <0.00005
Severely obese (BMI≥35 kg/m^2^)c, d	27.0 (70)	73.0 (189)	11.4, 0.0007	17.6, <0.00005
Overweight/mildly obese (28≤BMI<35 kg/m^2^)^d^	12.6 (37)	87.2 (252)		
Lean (BMI<25 kg/m^2^)^d^	15.6 (50)	84.4 (270)		
*Males*				
Severely obese (BMI≥35 kg/m^2^)^d^	10.0 (1)	90.0 (9)		
Overweight/mildly obese (28≤BMI<35 kg/m^2^)^d^	15.2 (31)	84.7 (173)		
Lean (BMI<25 kg/m^2^)^d^	19.5 (42)	80.3 (173)		

a Pediatric obesity clinic center patients.

b High school children.

c Adult obesity surgery clinic patients.

d SDPP and SMC participants.

### Adv36 is More Common in Severely Obese Females than Lean or Overweight/mildly Obese Adult Swedish Females and Men (Study 3b)

Positivity in Adv36 serology was more common among female patients with severe obesity (BMI≥35 kg/m^2^) that were referred to obesity surgery clinic in Stockholm between 2008 and 2010 than among lean healthy Stockholm/Uppsala (SDPP, SMC) females sampled 2002–2006 (Table 5). The increase in Adv36 positivity was 1.8-fold (relative risk) compared to lean (BMI<25 kg/m^2^) females (OR = 2.2, 95% CI = 1.4–3.2; χ^2^(1) = 13.5, p = 0.0002), and 2.3-fold compared to overweight/mildly obese (28≤BMI<35 kg/m^2^) females (OR = 2.7, 95% CI = 1.7–4.2; χ^2^(1) = 19.9, p<0.00005). Including also severely obese (BMI≥35 kg/m^2^) females not referred to the obesity clinic but enrolled in SDPP resulted the same association, 1.7-fold more prevalent Adv36 positivity compared to in lean females (OR = 2.0, 95% CI = 1.3–3.0; χ^2^(1) = 11.4, p = 0.0007) and 2.1-fold more prevalent Adv36 positivity compared to overweight/mildly obese females (OR = 2.5, 95% CI = 1.6–3.9; χ^2^(1) = 17.6, p<0.00005) (Table 5, Fig. 4B). No difference in prevalence of Adv36 positivity was detected between lean and overweight/mildly obese females (p>0.1). Due to a low number of severely obese males (n = 11), Adv36 association to severe obesity in males could not be analyzed. However, no difference in Adv36 positivity was found between females and males in lean and overweight/mildly obese groups (p>0.24), and no difference in prevalence of Adv36 positivity was detected among males between lean (BMI<25 kg/m^2^) and overweight/mildly obese (28≤BMI<35 kg/m^2^) (p>0.1) (Table 5, Fig. 4B). The age distribution differed between the severely obese females compared to the lean and overweight/mildly obese females and males (severely obese females: Mean ± SD: 42.0±10.6 years; overweight/obese females and males: 57.3±6.8 years; lean females and males: 57.3±6.5 years). However, this difference in age distribution did not influence the Adv36 association to female severe obesity, compared to BMI<35 kg/m^2^, since logistic regression with Adv36 status and age as covariates showed OR = 2.1 (95% CI: 1.3–3.5, p = 0.004) for Adv36 status.

Previous studies on Adv36 and obesity have mainly used BMI  = 30 kg/m^2^ as cutoff. Using BMI  = 30 kg/m^2^ as cutoff between ‘obese’ and ‘lean’ groups in this study the association between Adv36-positive serology and obesity was still present for females (OR = 1.5, χ^2^(1) = 5.6, p = 0.018). However, the statistical significance was lower compared to studying severe obesity (BMI≥35 kg/m^2^), as expected from the proportions listed in Table 5. Further, in the obese group (BMI BMI≥30 kg/m^2^) of females Adv36 positivity was associated with higher BMI (p = 0.014, Mann-Whitney U). In the obese females and males (BMI≥30 kg/m^2^) HOMA-IR, that reflects insulin resistance, tended to be lower for those Adv36-positive than those Adv36-negative using Adv36 status, cohort ID and sex as fixed factors and age and BMI as covariates (Adv36: *F* = 3.0, p = 0.084; cohort ID: *F* = 70.9, p<0.005; sex: p>0.1; age: p>0.1; BMI: *F* = 23.7, p<0.005; ANCOVA).

Having a high triglyceride level (>2.69 mmol/l) and/or being on pharmacological treatment for high circulating blood lipids (statins or fibrates) was less common among those Adv36 positive than among those Adv36 negative males and females (OR = 2.4, 95% CI: 1.2–4.9, χ^2^(1) = 9.6, p = 0.013). This was verified using logistic regression with Adv36 status, cohort ID, BMI-group, sex and age as covariates (Adv36: OR = 2.2, p = 0.039). However, quantitative levels of circulating triglycerides, cholesterol, glucose or insulin were not associated with Adv36 positivity in any of the three groups lean, overweight/mildly obese, or the severely obese group (p>0.1).

## Discussion

We show here that Adv36 positive serology, determined by an ELISA based on the Adv36 fiber protein, is associated with pediatric obesity (OR point estimate  = 1.6) and severe obesity in adult females (OR point estimate  = 2.0–2.7), with consequently approximately 1.5 to 2-fold increase in Adv36 positivity in cases compared to lean and overweight individuals. The individuals studied, including 424 children (10 to 18 years old), 1522 well-characterized adult females and males and 89 anonymous blood donors, were residing in central Sweden, Stockholm and Uppsala cities. The prevalence of Adv36 positivity in lean individuals residing in these areas increased from ∼7% in 1992–1998 to 15–20% in 2002–2006, and was stable until 2009. The rise in Adv36 positivity over time paralleled the increase seen in obesity in Sweden, which approximately doubled in prevalence between 1990 and 2005 [28]. The association with obesity was studied in samples collected in 2002–2010.

The increased prevalence of Adv36 positivity in obese children is in agreement with previously reported studies, using the serum neutralization assay (SNA) for Adv36 scoring, showing 2–3-fold increased prevalence of Adv36 positivity in children from San Diego (n_nonobese_ = 57 and n_obese_ = 67) and South Korea (n_nonobese_ = 59 and n_obese_ = 259) [17], [18]. Adv36 positivity was also associated with increased BMI among 84 obese children from South Korea [19]. The prevalence of Adv36 positivity of 29% in the Swedish obese children was similar to the 22–30% Adv36 positivity in the obese children in the three studies from San Diego and South Korea [17]–[19]. The samples from these studies were collected after 2005 with age range of the children (8 to 15–18 years) similar to that of the children in this Swedish report.

The increased prevalence of Adv36 positivity in Swedish severely obese females compared to non-severely obese individuals is in agreement with the report of a 3-fold increase in SNA-based Adv36 positivity in obese compared to lean adults from Wisconsin, Florida and New York (n_lean_ = 142 and n_obese_ = 360) sampled 1995–1999 (p<0.001). Similar to among children, ∼30% of the obese adults were Adv36 positive [13]. In addition, twins discordant for Adv36 status showed an association between Adv36 positivity and increased BMI [13]. Likewise, Adv36 positivity was 2-fold higher (prevalence 65%) in obese compared to non-obese Italian adults (n_obese_ = 68 and n_nonobese_ = 135) [15] and associated with greater BMI in Italian non-alcoholic fatty liver disease patients (n = 65) from Italy sampled 2007–2008 (n = 65) [16]. On the other hand, no association was found between BMI and Adv36 positivity in Dutch/Belgian adults (n = 509) with generally low prevalence of Adv36 positivity (5.5%) [29], nor among US military personnel (n_obese_ = 150 and n_lean_ = 150) showing an overall 36% Adv36 positivity [30]. Na et al found slightly higher prevalence of Adv36 antibodies (40% seropositivity) in overweight adults compared to lean adults (33% seropositivity) from South Korea [14].

That Adv36-obesity association in previous reports is less consistent in adults than in children maybe because other risk factors for overweight/obesity, such as life style, may have had a longer time to be expressed in adults than in children, and hence mask an Adv36 effect in adults. Also, children may have had less possibility to build up a protective immune response against Adv36 infection by exposure to other Adv36-cross reacting adenoviruses. Further, the antibody response to vaccination against virus has been shown to decrease with age [31] and BMI [32]–[34]; although there is no evidence for higher antibody titer against Adv36 in young compared to in old persons. However, it is possible that over time an Adv36 antibody titer in a person decreases to undetectable level. For example, a decline in antibody titer and T-cell response during the first year after antiviral vaccination was more pronounced in obese adults [35].

Nevertheless, we found Adv36 positivity to be associated with obesity in children, and to severe obesity in females, but not to mild obesity. Adv36 association with severe obesity in males was not investigated due to lack of samples.

Previous findings from *in vitro* studies of human tissue and *in vivo* studies in chicken, rodents and primates show that experimental infection with Adv36 induces fat accumulation in cells, preadipocyte proliferation, adipocyte differentiation, and body fat increase [4]–[12]. This supports the view that the Adv36-obesity association found in human serum samples reflects obesity as a result of the infection, rather than the possibility that the Adv36-obesity association reflects an obese state being more susceptible to Adv36 infection. A 2-fold increased risk for severe obesity by Adv36 with a prevalence of ∼15% in the control population, may be comparable to the increased risk of the currently known BMI-influencing genetic variations which together are reported to explain 1.5% of the variation of BMI according to genome-wide association analysis [36], [37].

We also found that high levels of circulating lipids, indicated by current treatment with statins or fibrates and/or a triglyceride level above the normal interval in untreated individuals, were less common in Adv36 positive compared to Adv36 negative adults. Accordingly, Adv36-associated obesity has been reported to have a good metabolic profile with low levels of blood lipids in animal [5] and in adult humans [13].

Further, we found a tendency, but not statistically significant (p = 0.084), for an association between Adv36 positivity and increased insulin sensitivity, measured as lower HOMA-IR in obese non-diabetic females and males (BMI≥30 kg/m^2^). Dhurandhar et al. [22] recently reported that natural Adv36 infection predicts better glycemic control in humans and showed additional data supporting that experimental Adv36 infection of animals increases adiposity and improves glycemic control. They have proposed that Adv36-induced increased glucose uptake by adipose tissue and skeletal muscle, increased adiponectin signaling, and reduced hepatic glucose output contribute to systemic glycemic control in Adv36- infected animals. In *in vitro* studies Adv36 induced peroxisome proliferator-activated receptor-γ and adipogenesis and concomitantly increased cellular glucose uptake via a Ras-mediated, phosphatidyl inositol 3-kinase (PI3K)-dependent up-regulation of glucose transporters [20], [21].

This paper is the first report of an ELISA assay to detect past infection with Adv36. Serial dilution of a known positive serum obtained from rabbits inoculated with killed Adv36 showed that the ELISA could detect Adv36 antibodies at concentrations 8 imes lower than the SNA could. This may explain that the ELISA scored positive for 36.9% of the the Adv36-SNA-seronegative samples among 367 human serum samples from United States, Korea and Finland. The specificity of the ELISA might be a concern as it picks up all antibodies directed against the Adv36 fiber protein fragment used as the ELISA plate coating antigen, and not only those neutralizing. However, the rate for positive Adv36-ELISA score in the 11 samples that were SNA-positive for Adv37 or Adv9 but SNA-negative for Adv36 was similar to that among the 293 Adv36-SNA negative samples with unknown Adv37/Adv9 score, suggesting that cross reactivity may be limited, although it needs to be investigated further in subjects (such as experimental animals) known to have been exposed only to Ad-viruses other than Adv36. Adv37, shown to induce obesity in chicken [38], and Adv9 belong to the same adenovirus-D subgroup as Adv36 and these three viruses are genomically substantially homologous. However, the CR1β, CR1γ, and fiber protein genes of Adv36 are quite divergent compared to those of other Adv-D viruses, such as Adv37 and Adv9; the Adv36 fiber protein shows only 60–70% homology to the other Adv-D viruses sequenced [3]. The ELISA scored positive for 74.3% of the samples positive in the Adv36-SNA. Since the SNA scores signal from all neutralizing Adv36 antibodies in the serum, whereas the ELISA measures only antibodies to a fragment of the fiber protein, the ELISA may fail to detect some samples from true prior Adv36 infection. Hence, the rabbit serum experiments showed that the ELISA detects lower titers of antibodies to Adv36, but the concordance test in human samples showed that not all persons with neutralizing antibodies to Adv36 have those antibodies detectable by the ELISA. However, the fact that we detect association between the Adv36-ELISA-signal and the clinical phenotypes of obesity and insulin sensitivity similar to the associations with these variables found in previous studies [19], [22] strongly suggests that the Adv36-ELISA indeed measures prior Adv36 infection. In addition, our data show that the Adv36-SNA has a higher uncertainty rate, with about twice the rate of equivocal results compared to the ELISA.

There are some limitations in this study. The sample size was small (i) for testing cross reactivity between Adv36, Adv37 and Adv9, (ii) for estimating Adv36 prevalence in samples collected in Stockholm 1992–1998, (iii) of severely obese adult males. The age distribution was different between obese/overweight children and lean children, however, age, sex and BMI SDS was controlled for in the analysis. For 29% of the obese/overweight children and the adults studied there was no information available regarding weight reduction efforts prior to sampling. There was no blood chemistry data for the lean children and the SMC adult females. Data on blood lipid levels were missing for a considerable number of SDPP adult individuals.

To conclude, we developed a rapid ELISA and show that Adv36 infection is associated with pediatric obesity, severe obesity in adult females and lower levels of blood lipids, and that those infected have a tendency for increased insulin sensitivity in non-diabetic Swedish individuals. These findings agree with previous studies *in vitro*, in experimental animal and most human population studies. We also show a rising prevalence of Adv36 infection in Sweden from the period of 1992 to 2009, the first longitudinal data suggesting an increasing prevalence of Adv36 infection in parallel to an increase in the prevalence of obesity. These findings address comments in a recent review arguing for the need of further epidemiological studies to establish the link between viral infection and obesity in man [39]. The importance of a rapid Adv36 detection assay that may be used clinically is demonstrated by the study of Trovato et al [40] which demonstrated that a positive clinical response of non-alcoholic fatty liver disease to nutritional treatment was enhanced in Adv36-positive compared to Adv36-negative patients. This study suggests that detection of Adv36 in patients may guide the selection of treatment. Further studies on Adv36 association with metabolic states in large population cohorts are justified.

## Supporting Information

Information S1 **Detailed descriptions of serum neutralization assay (SNA) for viral antibodies and ELISA for antibodies against Adv36.**
(DOC)
