# Supplementary material for: Adenovirus-36 Is Associated with Obesity in Children and Adults in Sweden as Determined by Rapid ELISA
Source: PLoS One. 2012 Jul 27;7(7):e41652. doi: 10.1371/journal.pone.0041652 (PMC3407196; doi:10.1371/journal.pone.0041652)
Supplement: Information S1 — Detailed descriptions of serum neutralization assay (SNA) for viral antibodies and ELISA for antibodies against Adv36. (DOC) [file pone.0041652.s001.doc]

**S1. Supporting information on Methods**

Serum neutralization assay (SNA) for viral antibodies

Positive control serum samples were produced by inoculating three rabbits with killed Adv36 emulsified in Freund’s adjuvant via intramuscular injection and collection of blood 29 days post-injection (Obetech, Richmond,VA). Human serum samples (n=367) from the United States, Korea and Finland were used to compare the Adv36-ELISA to the Adv36-serum neutralization assay (SNA). The SNAs were performed, as described previously [4, 5]. In brief, human sera were serially diluted (two-fold) from 1:2 to 1:128 in 96-well plates. For each virus tested, a total of 100 TCID_50_ (50% Tissue Culture Infective Dose) of the respective adenovirus work stock (Adv36, Adv37, Adv9) (American Type Culture Collection, Manassas, VA) was added to each of the wells. A549 cells (ATCC, Manassas, VA) were added to wells after 1 h of incubation at 37°C. Each serum sample and the positive rabbit control serum were run in duplicate with additional appropriate controls [4, 5]. Plates were incubated at 37°C for 11 days and the presence of cytopathic effect (CPE) was noted. Serum samples without CPE in dilutions of 1:8 or higher were considered positive. Disparate readings with one reading above and one below the cutoff value were considered equivocal.

ELISA for antibodies against Adv36

The investigation is presented according to the Standards for the Reporting of Diagnostic Accuracy (STARD) (http://stard-statement.org/). The analyses were performed by researcher blinded to any phenotypic and SNA data. 96-well ELISA microplates (Costar, Corning, NY, USA; alternatively Perkin Elmer Spectra plate-96 HB, Waltham, MA) were coated with 100 ul of coating buffer consisting of 0.05 M Na_2_CO_3_ and 0.05 M NaHCO_3_ adjusted to pH 9.6 and coating protein (final concentration 10 ug/ml) consisting of recombinant Adv36 fiber protein fragment fused with maltose binding protein (Obetech, Richmond, VA). Plates were sealed with sealing film and incubated overnight at 4°C. The plates were uncovered and washed 10 times with deionized water at room temperature (for in total ~1.5 min) to remove excess coating protein. A blocking protein buffer was prepared with 20 mg/ml of bovine serum albumin (minimum 98% electrophoresis grade, Sigma- Aldrich, St. Louis, MO, USA) dissolved in phosphate buffered saline at pH 7.4 (Invitrogen, NY, USA). 100 ul of blocking buffer was pipetted into each well and the plate was sealed and incubated at 25°C and 300 rpm (Shaker Thermostar, BMG Labtech, Ortenberg, Germany) for 1 h. (Alternatively, incubation with blocking buffer can be at 4°C overnight.) The plates were uncovered and washed 10 times with deionized water at room temperature (in total ~1.5 min) to remove excess blocking protein. A mixture of 5 µl of horse radish protein (HRP conjugated coating protein, Obetech, Richmond, VA) and 125 µl of the competitor protein recombinant maltose binding protein 2.5 mg/ml (MBP, Obetech, Richmond, VA, final concentration 62.5 µg/ml), in 5 ml of blocking buffer was prepared. Using aseptic technique in a sterile tissue culture hood at room temperature, 50 µl of HRP-MBP mix and 50 µl of serum from the subject was added to each well and mixed thoroughly. Each 96-well plate had one positive serum control (50 µl positive rabbit control serum (diluted 1:80) (Obetech, Richmond, VA)), one background control (50 µl of blocking buffer), and at least one control serum known to be at the cutoff value for positive ELISA score being human serum (50 µl) and rabbit serum (50 µl of dilution 1:1280). The plate was sealed and incubated at 25°C and 300 rpm for 1 h, then uncovered and washed 20-25 times with deionized water in room temperature (for in total ~3 min). 50 µl of TMB substrate solution (3,3’,5,5’ – tetramethylbenzidine, Thermo Scientific 34028, Rockford, IL) at room temperature was added to each well, the plate sealed, and incubated at 25°C and 300 rpm for ~25 min. 100 µl of 1M HCl was used to stop the reaction. The plate was read at a wavelength of 450 nm using a microplate reader (Multiscan EX, Thermo Scientific, Rockford, IL, USA; alternatively Bio-Tek Power wave XS microplate reader, Winooski, VT) within 20 minutes after stopping the reaction. A 12-channel multipipet was used except for when adding the serum samples. For washing, an 8-channel plate washer was used. All samples were run in duplicate. The positive serum control must have an optical density (OD_450_) reading greater than 1.5, and the background control must have a reading below 0.06 or the assay was invalid. The negative human control serum should be within OD_450_ ± 0.05 from its reference value. ELISA cutoff for positive score was based on analysis of human serum samples with known Adv36-SNA status (to avoid bias in the ELISA-SNA comparison reported here, these serum samples used to determine cut-off point were not used for the ELISA-SNA comparison). Thereafter, the OD_450_ reading from the 1:1280 dilution of the positive rabbit serum was used as cutoff marker for positive ELISA score since it gave a signal corresponding to the defined ELISA cutoff. An OD_450_ equal or greater than this value in both duplicates was scored as a positive assay for Adv36 antibodies. A reading of less than this value in both duplicates was scored negative. Disparate readings with one reading above and one below the cutoff value were considered equivocal and the sample was reanalyzed in duplicate. A sample with repeat analyses was considered to be positive if >50% of the replicates were positive with an OD_450_ higher than the cutoff value. Hence, data from a reanalyzed serum sample were used only if the reanalysis provided consistency between the duplicates. Human serum samples (n=367) from United States, Korea and Finland were used to compare the Adv36-ELISA scores to the Ad36-SNA scores. These samples were not reanalyzed.
